# Clinical Reasoning in the Real World Is Mediated by Bounded Rationality: Implications for Diagnostic Clinical Practice Guidelines

**DOI:** 10.1371/journal.pone.0010265

**Published:** 2010-04-20

**Authors:** Ana Paula Ribeiro Bonilauri Ferreira, Rodrigo Fernando Ferreira, Dimple Rajgor, Jatin Shah, Andrea Menezes, Ricardo Pietrobon

**Affiliations:** 1 Univille, Joinville, Brazil; 2 Duke-NUS Graduate Medical School, Singapore, Singapore; 3 Hospital Angelina Caron, Curitiba, Brazil; 4 Duke University Health System, Durham, North Carolina, United States of America; 5 Research on Research group, Duke University, Durham, North Carolina, United States of America; Norwegian Knowledge Centre for the Health Services; University of Oslo, Norway

## Abstract

**Background:**

Little is known about the reasoning mechanisms used by physicians in decision-making and how this compares to diagnostic clinical practice guidelines. We explored the clinical reasoning process in a real life environment.

**Method:**

This is a qualitative study evaluating transcriptions of sixteen physicians' reasoning during appointments with patients, clinical discussions between specialists, and personal interviews with physicians affiliated to a hospital in Brazil.

**Results:**

Four main themes were identified: simple and robust heuristics, extensive use of social environment rationality, attempts to prove diagnostic and therapeutic hypothesis while refuting potential contradictions using positive test strategy, and reaching the saturation point. Physicians constantly attempted to prove their initial hypothesis while trying to refute any contradictions. While social environment rationality was the main factor in the determination of all steps of the clinical reasoning process, factors such as referral letters and number of contradictions associated with the initial hypothesis had influence on physicians' confidence and determination of the threshold to reach a final decision.

**Discussion:**

Physicians rely on simple heuristics associated with environmental factors. This model allows for robustness, simplicity, and cognitive energy saving. Since this model does not fit into current diagnostic clinical practice guidelines, we make some propositions to help its integration.

## Introduction

Clinicians make numerous diagnostic decisions that affect patient's well-being and although most of the time these decisions are correct, sometimes they can be fatal [1]. Clinicians are often advised to base their decisions on clinical practice guidelines that are informed by evidence-based medicine (EBM) [2]. However, non-adherence to clinical practice guidelines is a perennial problem extensively documented in literature [3]–[6]. While the literature identifies multiple reasons for this failure like, differences in practice settings, expectations and behavior of the patient, mode of implementation, display of guidelines, we believe that the central problem is a disconnect between the assumptions about how clinical decisions are made and the actual practice of decision making. Precisely stating, clinical decisions are assumed to be far more rational and deductive than they actually are, which can lead clinical practice guidelines to fall out of sync with clinician's decision realities. Despite having extensive literature in the field of clinical decision-making, there remains a paucity of formal studies to understand this decision making process.

Clinical practice guideline advocates consider that medical decision making should be based on an explicit, quantitative and systematic approach [7]. Conversely, literature on clinical reasoning has demonstrated that clinicians are not able to follow purely rational, highly formal and logical thinking due to inherent limitations of human cognitive capacity that prevents storage, as well as processing of multiple rules in short periods of time [8]–[9]. The use of diagnostic clinical practice guidelines (DCPGs) can sometimes be challenging and may be with limited effectiveness, mainly in cases of complex symptoms such as syncope [10], [11], [12] or complex diagnosis such as disease syndromes having multiple criteria. Decision trees used in DCPGs are often more focused on accuracy than usability and their complexity may stop clinicians from using them, even when there is evidence of their benefit [13]. In clinical environments, the risks and costs of making optimal decisions are often far greater than those of satisfactory ones [14]. Thus, in addition to explicit quantitative data, clinicians also rely on social environmental information (for example: depending on how patients behave and what they expect) [5]. They also use an adaptive approach that incorporates new information as it becomes available over time. This signals the frequent use of heuristics in clinical decision making wherein clinicians, like all decision-makers, make the best decisions they can with the limited information available [15]–[16].

Research on the widespread use of heuristics has typically focused on how biases can lead to reasoning flaws, for example: if you have already seen several cases of a disease you can have a tendency to diagnose the same disease not taking into consideration less usual diagnosis; another example is the collection of more data than needed due to conservatism. These studies led to erroneous conclusions, overlooking how successful, heuristic based reasoning can be [17]. Although there have been studies examining the use of heuristics in decision making, most of them are not related to the clinical environment. The few which are related to clinical decision making are laboratory experiments based on surveys, questionnaires and interviews. In addition, these studies present multiple theories that are highly debated. For example, while there are theories proposing that the clinicians use a deductive or elimination approach in making diagnostic decisions, the bounded rationality theory emphasizes the importance of including environmental factors in clinical decision making [18], [19], [20]. One of the few real world studies, published by Bruyninckx et al (2009), demonstrated the importance of environmental factors in the referral decision process. Natural setting evaluation is important to fully understand clinical decision making [21]. To our knowledge, there are few previous studies evaluating the clinical reasoning process in the clinical environment.

Thus, the aim of this qualitative study is to explore the clinical reasoning process in a real practice environment in order to understand how physicians make decisions in their daily practice and relate these findings to DCPG implementation.

## Methods

### Subjects

Sixteen physicians gave written informed consent and agreed to participate in this study. We conducted the study at Hospital Angelina Caron, a medium-size hospital in Brazil which is a referral center, performing wide range of complex, as well as routine diagnostic and therapeutic procedures. The study was approved by Hospital Angelina Caron's Ethics Committee.

### Sampling

A convenience sampling scheme called snowball sampling [22] was used for selection of participants. Following this sampling method, we did not use statistical sampling but rather guided our sampling by the analysis performed after each interview in that new findings guided the choice of other clinicians and clinical situations that would confirm or reject a given theme. Accordingly, we asked the participants of the study to recommend to us their colleagues or friends, whom we could approach to check their willingness and subsequent participation in the study.

### Data collection

Data was collected using voice recordings from three different activities:

#### 1. Regular clinical encounters between participating physicians and patients

All clinical appointments were voice recorded with prior written informed consent from patient and physician. All these appointments were outpatient and our research coordinators acted just as observers. Additional notes were made about the office environment, physician's attitude during the appointment, such as expressions of reflection or concern, and physician's notes in the medical chart that could provide additional insight about the clinical reasoning process.

#### 2. Clinical discussions between specialists

When further insight was needed to address complex cases, physicians consulted with colleagues in other specialties. These meetings occurred frequently in the wards. When a physician made a comment about the need for such a meeting, our research coordinator attempted to attend this meeting whenever possible. If multiple discussions occurred over time, every effort was made to attend all of them and follow the case longitudinally until a final diagnostic or therapeutic decision had been made. These encounters were also voice recorded.

#### 3. Personal interviews with participating physicians

We conducted individual interviews with participating physicians after analyzing the transcriptions for the physician-patient and physician-physician discussions. All interviews were conducted at the physician's office. The interviews were considered as a fundamental instrument to deepen understanding, usually by obtaining clarifications about specific events that happened during clinical interviews, asking for clarification on thought processes occurring during the clinical encounter.

### Transcription

In order to improve accuracy, all discussions were transcribed verbatim by the research coordinator who collected the data (APM) within seven days from data collection. Physician and patient identifiers were removed from the transcript to ensure confidentiality. Notes and impressions obtained during the interview were added to the original transcription as comments.

### Data analysis

In the analysis, we used a grounded theory approach to describe occurrences as they pertained to the individual; without recourse to previous theory, deductions, or assumptions from other disciplines [23]. Prior to this study's commencement, none of the investigators had a preconceived hypothesis to explain our findings. To interpret the phenomena surrounding our findings, we relied primarily on our own judgment. Within this framework, we analyzed each transcription following three standardized steps: (1) Obtained a feeling of the entire transcription through multiple readings; (2) Identified meaning units and extracted the ones that were relevant for the clinical reasoning process; and (3) Integrated different meaning units into emerging themes that were compared against previous interviews [24]. This process was conducted after each interview, and re-emerging themes were then tested in subsequent interviews for their presence or absence. Physician's notes were used in the process of triangulation, therefore confirming or rejecting some of our observations. Thereafter, we explored the roles of these emerging themes in the clinical reasoning process.

Transcriptions were coded line by line using a combination of hand coding and the N6 software [25]. The senior author (RP) initially coded each transcription, which was later checked and discussed with two other independent researchers[DR, JS]. We did not measure observer reliability and all disagreements were resolved through consensus. Data saturation was defined whereby consistent themes emerged across encounters, physicians and different environments after successive interviews. This was achieved by the time data from 16 physicians which included 20 patient appointments, 16 clinical discussions, and 10 personal interviews, were collected.

## Results

### Emerging Themes

**Figure 1:**
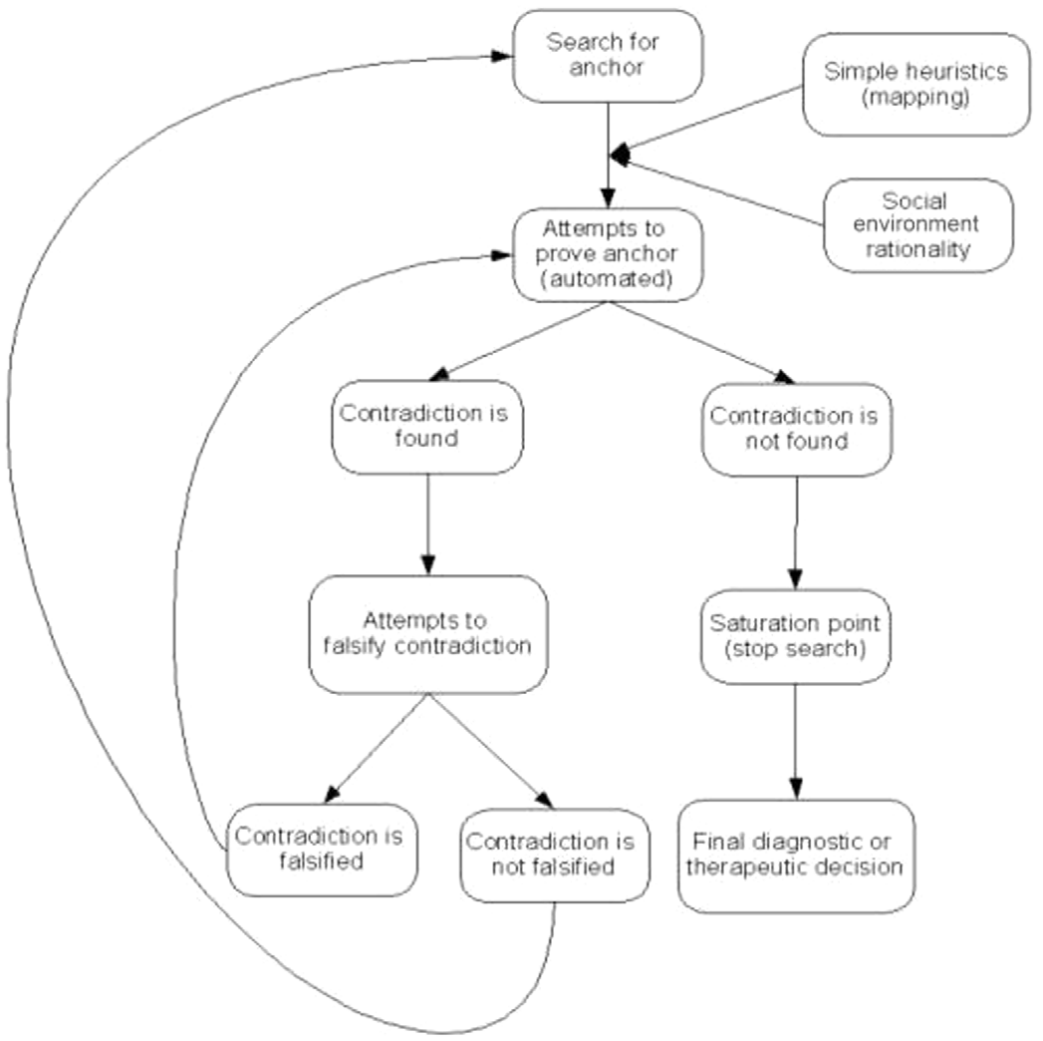
Pathway for clinical reasoning process.

After analysis of the initial four transcriptions, we observed that routine clinical appointments involved unchanging diagnostic or therapeutic hypotheses. In contrast, complex clinical cases and referrals required more elaborate reasoning and, subsequently only complex cases were selected. Based on the analysis of these cases. we found four related emerging themes that described the clinical reasoning phenomena: 1. Simple and robust heuristics, 2. Social environment rationality, 3. Attempts to prove hypothesis and refute contradiction and 4. Saturation point. These emerging themes can be interlinked in different manners at different phases of the clinical reasoning process (Figure 1). Therefore, the emerging themes and sequence will be described separately. All emerging themes can be described and aggregated around (1) the search for a hypothesis for diagnostic or therapeutic choice, and (2) decreasing the amount of cognitive effort and time necessary to reach the hypothesis. Each of the emerging themes will be described in relation to these two factors. The characteristics of participant physicians are displayed in Table 1. To facilitate the interpretation of our study findings, a taxonomy was created which is listed in List S1.

**Table 1:** Characteristics of participating physicians (gender and specialization X years of practice).

	1–10 years		10–20 years		>20 years	
	n	%	N	%	n	%
Female Cardiologist	1	6,25				
Female Pediatrician	2	12,5	1	6,25		
Female Intensivist	1	6,25				
Male Cardiac Surgeon	2	12,5	1	6,25		
Male Cardiologist					1	6,25
Male Pediatric Cardiologist	1	6,25				
Male Interventional Cardiologist	2	12,5			1	6,25
Male Pneumologist			1	6,25		
Male Nephrologist					2	12,5

### Simple and robust heuristics

Physicians always began with a series of exploratory questions about past, personal, and family medical history, as well as current symptoms. Although slight variations were found, questions tended to be standardized within specialties. For example, while pediatricians would always ask about conditions related to the birth of the child, nephrologists had a series of standard questions about the aspects of the urine. These questions were exploratory in nature and tended not to focus on any specific hypothesis. Some quotes on these exploratory questions are as follows: “Tell me about diseases you had when you were a child”, “What is the reason you came here”, and “Do you have any allergies?”. When asked about the reason for the general heuristic, physicians repeatedly mentioned that a standard set of questions resulted in consistent results and would also save cognitive energy. A pediatrician mentioned: “Asking these questions gives me a direction…allows me to find some clues on the diagnosis….Not using them would increase the amount of work because I would have to start with questions…that would probably not lead me anywhere.” Physicians used these general questions as a way to find one or more signs or symptoms that would trigger a simple and robust heuristic, or a clue that would define a diagnosis and subsequent treatment. This simple and robust heuristic was applicable to a wide range of conditions while maintaining its validity.

When this trigger was activated, physicians would stop exploring and start on a fixed path looking for the confirmation rather than falsification of a diagnosis and subsequent treatment plan. Although a hypothesis could have a multitude of clinical signs and/or symptoms attached to it, physicians believed that a group of characteristics constituted a pattern representative of an individual hypothesis. For example, a female pediatrician, 10 years in practice was asked for an example of how she usually makes a diagnosis said that “Well … we [referring to pediatricians in general] usually already have in mind what is necessary and then try to find exactly what we would expect.” Another example, while abdominal pain alone was not found to be associated with a diagnosis, a pediatrician mentioned that “pain in the lower right quadrant, some fever… and this sign [shows the Bloomberg sign (where she elicits pain through a maneuver of slow compression and sudden decompression) at the McBurney's point] would suggest appendicitis.” At this point, the physician recognized the pattern, stopped using general heuristics and focused on hypothesis-specific heuristics. When asked about the reason for the focus, physicians mentioned that they were already confident about the hypothesis and that further investigation would lead to a waste of time. From this point on, physicians looked for cues that would confirm the hypothesis. This search continued until the saturation point was reached.

### Social environment rationality

Social environment rationality was used during the whole reasoning process and was represented in two principal ways. First, during the general heuristics phase, physicians commonly relied on information from the social environment. These environmental sources included referral letters and previous information from medical charts, comments from assistant physicians, or clinical discussions with other physicians. During personal interviews, physicians reported this kind of information as “concentrated or distilled”. According to a nephrologist, “you can gather a lot of information by talking to the family [member]”; while an interventional cardiologist felt that “letters from the family practitioner will frequently provide the additional information we need… to reach a diagnosis… in association with our examination findings.”

Second, information from the social environment helped the physician to determine the level of complexity to be used in his chosen heuristics. When the information from the social environment pointed to a routine case (something seen by the physician several times a week), simple specific-hypothesis heuristics were selected. In contrast, environmental information that pointed to a complex case (a case that was diagnosed only few times a year) involving multiple previous attempts of diagnosis or therapeutics, or a patient that was referred by a colleague, physicians applied more complex and exhaustive heuristics. For example, a male pediatric cardiologist, 10 years in practice received a patient referred by a general pediatrician with a diagnostic hypothesis of a congenital cardiac anomaly. Although history and physical examination were normal, he continued the investigation with additional exams. He said “You see, the exam is apparently normal, with nothing that calls my attention… but we need to continue the investigation now with an EKG and echocardiogram to evaluate the heart of the baby.”

Environmental information pointing to a higher degree of complexity also resulted in hypothesis-specific heuristics that led physicians to suspend judgment in a greater number of cases. In such cases, final clinical decisions were confirmed only after additional exams were obtained and/or further discussions with peers. In other words, perceived complexity obtained from social environment led to a cautionary attitude in decision making. According to an interventional cardiologist, “When we start with a complex story from a referring physician… that we know is a good doctor… we should take extra care to make our diagnosis since there might be a lot of factors going on.”

### Attempts to prove hypothesis and refute contradiction

Once an initial pattern was recognized and a hypothesis established, physicians moved from a general heuristic to a hypothesis-specific heuristic with a positive test strategy. Unlike researchers who use a statistical hypothesis testing strategy that aims to refute an initial hypothesis, physicians use a strategy focused on confirming their initial hypothesis. Although contradictory to the perceived image of a clinician always looking for differential diagnosis, this heuristic seems to provide an economical way of formulating a diagnosis or therapeutic plan. Besides it would be cumbersome for physicians to constantly doubt their own initial hypotheses in search of alternative diagnoses or therapeutic approaches. According to a pediatrician “we would not be able to finish our clinic if we [were to] think about differential diagnosis all day… and this works [referring to the method of not thinking about differential diagnosis all day].”

When information contradicting the initial hypothesis emerged, its sources were always the patient or the physician's peers, not the physician himself. The physician would first attempt to prove the initial hypothesis by refuting the contradicting information rather than immediately moving to an alternative hypothesis. For example a male interventional cardiologist, 10 years in practice, when asked by the researcher about his attitudes when the symptoms referred by a patient do no match a diagnosis said: “… what one does is to explore the symptoms to check if what he [referring to the patient] reports as his symptoms is or is not something … many times what is really dear for us is not [for him] … sometimes he may say that he is short of breath, but is really not dyspnea.” Another example is, a male cardiac surgeon with 15 years in practice, that during an appointment with a low educational status patient who referred “cramps” in his legs disregarded the patient's complaints since they were not in agreement with his hypothesis. Only, when physicians could not refute the contradicting information, would they begin to explore alternative hypotheses and use more complex heuristics. Contradicting information was most prevalent in complex cases which were discussed with peers. At that time, these peers raised contradictions and sometimes alternative hypotheses (i.e., differential diagnosis) that enhanced the quality of the final diagnosis. Contradictions raised by peers presented a greater weight and were harder to refute than contradictions raised by the patient. It should be emphasized that peers were consulted only for difficult cases, which again points to a parsimony and attempt to save cognitive energy while handling the clinical reasoning process. Simple cases are handled by a simple hypothesis-specific heuristic that can only be contradicted by patient information, whereas complex cases could be contradicted by both patients and peers. In summary, different case complexities required heuristics with a different level of exposure to contradiction.

### Saturation point

Once a hypothesis was established and either the hypothesis-specific heuristic had no apparent contradiction or all contradictions had been refuted, the physician went to a saturation point where the case was deemed to be resolved. This saturation point was at most times reached at the same point as the point of no contradictions, but below, we point out two common factors that could delay its reach although all contradictions were resolved.

First, the presence of multiple contradictions tended to reduce physician's confidence and, as such, increased their threshold for saturation. In other words, if while evaluating a patient, the physician had several points that initially “didn't seem right” (according to a cardiac surgeon), but that were later refuted to confirm the hypothesis, the hypothesis was weakened and its acceptance was delayed. It was unclear why this occurred, but apparently the physician's confidence in the overall information provided by the patient or laboratory exams decreased substantially in spite of successful attempts to refute all contradictions. When asked why this occurred, a pediatrician said that “even though there is nothing against my diagnosis, there were just too many ‘almost wrong’ things… going on…I just don't feel good about this diagnosis.”

Second, social environmental factors such as the opinion of peers seemed to play a crucial role in the determination of a threshold for acceptance of a hypothesis. Since contradictions raised by peers seemed to have a greater weight than those raised by patients, finding contradiction among peers even though the clinician successfully refuted them, frequently led to a suspension of the final decision. In these circumstances, a saturation point was reached without a diagnosis and the heuristic was postponed while waiting for further information in the form of additional exams, additional peer opinion, or simply for the appearance of new evidence as the disease progressed.

## Discussion

We found that clinicians frequently use simple heuristics in the process of clinical decision making. In this discussion, we will argue that these simple heuristics are in contrast with the way criteria are defined in diagnostic clinical practice guidelines (DCPGs). Simple heuristics were divided into general and hypothesis-specific heuristics. Questions based on general heuristics were exploratory and were used to save time and effort in the process. Use of these heuristics led to the discovery of cues that guided the formulation of a hypothesis. At this point, hypothesis-specific heuristics were employed until a saturation point was reached. Social environment rationality represented another way of decreasing the amount of cognitive energy used in the search of a hypothesis and, afterwards, in its confirmation. This approach helped physicians define a diagnosis and determine a saturation point, sometimes being the most important factor in delaying the final diagnosis.

When trying to make diagnostic or therapeutic decisions, physicians based their clinical reasoning on simple and robust heuristics rather than relying on a powerful memory to remember lists from diagnostic criteria that are typical from DCPGs. Our findings are in accordance with the theory of bounded rationality which provides plausible explanations for physician's ability to make intelligent choices quickly, in an economic manner related to cognitive energy, and with minimum necessary information by exploiting the way information is structured in their environment [9]. Aided by information obtained from the environment, bounded rationality proposes that simple heuristics perform comparably or even outperform more complex algorithms, particularly when generalizing to more complex cases [26].

We observed the use of “simple” heuristics instead of rule based decision trees (the ones usually stated in DCPGs). Besides being simple, these heuristics were also “robust” since they could be applied to a wide range of clinical situations. In other words, simplicity leads to robustness. The notion of bounded rationality also promotes a vision of clinical reasoning based on three premises. First, it assumes that due to time, knowledge, and memory constrains, physicians base their clinical decisions on simple heuristics that are at the same time robust and precise. Second, it offers solutions that are domain-specific rather than general. Bounded rationality provides heuristics that are composed of cognitive and environmental models that can be a part of multiple heuristics and can allow for the composition of new heuristics. Third, the success of the bounded rationality strategies is related to its degree of adaptation to the structure of environments, both physical and social [9], [27]. The adaptation and selection of strategies from a broad repertoire is based on a cost-benefit approach which states, the less cognitive effort the better [21]. Therefore, our findings disagree with previous studies in which clinical reasoning has been considered to be unbounded, or unlimited by constraints such as time and ability to process a massive set of information [19], [28]. This shows a contrast in the way decisions are taken in the real world and the way DCPGs are presented to guide decisions. While real life follows simple strategies, DCPGs can be complex leading to lower compliance and adherence [13]. For example, they involve decision trees that may deter clinicians from adopting and adhering to them in real life scenarios [13]. A better approach to clinical decision making could involve its marriage with an electronic medical record where a multitude of guidelines would be compared with a patient's condition and treatment strategy. In such a case, real time analysis of patient data against the recommendations of a guideline would be presented in a simple graphical format that would facilitate a clinician's decision making process. Such a tool would make guidelines more user-friendly while better reflecting real life scenarios [29].

Information gathered from the environment (social environment rationality) was represented in our study as comments from other physicians, referral letters and medical charts. This environment-based information was important in defining not only the appropriate heuristics to be used, but also the saturation point. While receiving this information, clinicians looked for high validity cues. Although the strategy of ‘searching cues from the environment’ is important, in some instances, too much information can come from it leading to computational needs beyond human capabilities. Furthermore, it has been previously noted that there is a risk of making errors when thinking that a particular patient is fundamentally different from the other, similar patients [9]. Some EBM advocates also postulate that the use of the environmental data is misleading [30]. We agree that environmental factors and cues are limited by the ability of the observer to register, understand and incorporate them in clinical decision making. However, when utilized correctly and judiciously, they can contribute to better decisions at a lesser cost. [17], [31], [32]. Although, some environmental factors maybe included in DCPGs, inclusion of all the factors poses a limitation. Efforts should be directed at understanding certain common and general aspects of influence of environment, such as referrals, charts and notes. The incorporation of the most common aspects gathered from the environment in DCPGs would allow for shortcuts, where some steps could be skipped without the risk of losing accuracy.

During the use of hypothesis-specific heuristics, clinicians directed their efforts toward a diagnosis that confirmed their hypothesis and refuted all possible contradictions. This finding is in contrast with the classical idea of clinicians frequently attempting to refute their initial diagnosis through differential diagnoses [33], or clinicians trying to rule out potentially serious diagnosis [34]. Some of the work in this area acknowledges these findings as confirmation bias and suggest that directing data collection to the confirmation of hypothesis can lead to sustaining an inappropriate, and most of the time, premature hypothesis, neglecting important alternative data [9], [21], [33]. On the other hand, there are authors who encourage the use of a positive test strategy (i.e. focused on confirming rather than rejecting the hypothesis) since the replication of cases consistent with the hypothesis tested have a good chance of achieving the expected result [33]. Our findings were consistent with the later view, since while clinicians do not look for a contradiction themselves, their action, when one is pointed out by the patient or environment, is to try to refute it, sustaining their initial hypothesis. This seems to be related to the additional effort that would be necessary to re-start the process of looking for another hypothesis. This phenomenon again points to bounded rationality which looks for satisfying and not optimizing the costs of giving up of a hypothesis without trying to sustain it, as this search could exceed the benefits [35]. If the same process is compared with the DCPGs for final diagnostic or therapeutic conclusions, DCPGs would list several elimination steps for differential diagnosis. This would make the whole process cumbersome and cost driven, not only for the physician but also for the patient. The reason for this complexity lies in the nature of the DCPGs, which are disease specific and not patient specific. A clinician may encounter different types of patients with different and sometimes even multiple diseases in the clinic [36]. Thus, a physician's judgment is based on several other factors pertinent to that specific patient and environment and not only the ones suggested by DCPGs. The DCPGs could rarely provide recommendations on these aspects. Following any disease specific guidelines can always put the physician in dilemma about reaching a conclusion for confirming the hypothesis about the diagnosis. In the given scenario, physician's tacit knowledge and contradictions put forth by the environment and by the patient are also important.

‘Saturation point’ was defined as the moment when physicians decide to stop using heuristics. It could be reached either because physicians confirmed a hypothesis or because they halted the process until further discussion was held with peers or they could wait for further evolution of their patients' condition. This method of limiting and stopping the search as soon as they felt satisfied with the decision is part of Simon & Selteńs models of bounded rationality [37], [38]. As soon as the physician using hypothesis-specific heuristics with a positive test strategy found a pattern, the process was stopped and a diagnosis was reached. This process could be delayed when there were many cues to the diagnosis that needed to be refuted. The effort to refute cues lead to a reduction in physician's confidence and a rise in uncertainty levels. Contradictions from the environment, particularly contradicting peer opinion led to the same result. Confidence in own judgment is also found to be an important factor in decision making whereby employing heuristics [39], [40]. In our study, confidence was a chief factor for establishing the saturation point, but it certainly was not the only component. For instance, time pressure, although not observed in this study, must be taken into account, and further study should be conducted in this area [35]. DCPGs should try to limit multiple steps in order to reach a conclusion. This should happen with prior, active involvement of physicians' right at the developmental stage of the DCPGs. A probable solution may be obtaining consensus of the physicians and giving attention to the adoption of the guidelines right at the time of DCPG development process.

The use of a convenience sampling method and presence of researchers during the process of clinical decision making might have an impact on our findings and thus represent a limitation of our study. Yet another limitation was the difficulty to follow-up all physician encounters with colleagues, since these encounters occurred by chance, without a previous schedule and although the researcher attempted to attend all meetings there were occasions when this was not possible. In certain cases, presence of a nurse might have had an effect on the course and content of discussion. However, this was not taken into account in the current study. The study represents the decision making process utilized by a small sample of the clinician population. Our results are not generalizable but serve the purpose of understanding and evaluating the real world scenario.

DCPGs are primarily based on differential diagnosis which involves a stepwise elimination of multiple hypotheses following an evidence based strategy. Thus, in order to attain saturation and reach a conclusion following the DCPGs, cost and time for decision making must be affordable. Although, DCPGs as standard of practice are important, they should constitute as bare minimum requirements as possible, thus ensuring adherence. Alternatively, backward design can be used in structuring DCPGs wherein the diagnosis may start with the most relevant and possible options which can then be either confirmed or refuted. As a next step, subsequent diagnostic test interventions can be listed in the order of decreasing possibilities.

We believe that the current DCPGs do not follow a backward design approach, probably because they need to reflect a standard approach that can be utilized in routine clinical practice. In routine clinical practice, each patient could present with several signs and symptoms along with co-morbid conditions and ongoing medications. DCPGs can not encompass all this information as it would make them complex. They have to be standard following a bottom up approach. In such cases, heuristics, based on different permutations and combination of available signs, symptoms and other data, guide the clinician to arrive at a hypothesis, get views from peers, try to refute the ones not aligning, order a diagnostic test and get the hypothesis confirmed. Another important point is that foolproof diagnostic methods with high sensitivity and specificity do not exist for several diseases. Therefore, in spite of ordering diagnostic tests, it is not sure that they will lead to confirmation of diagnosis. For example, patient may have specific symptoms pointing towards a diagnosis but the diagnostic test may return a negative result. In such cases, heuristics supersede guidelines. Additionally, backward design may not work in DCPGs because they need to be simple and general enough so that they can be used by clinicians with different sets of experience including novice clinicians. For an inexperienced clinician, based on the available information about a patient, it might be difficult to start with the most relevant diagnosis as in case of backward design approach followed in heuristics, rather they would prefer the differential diagnosis as stated in the DCPGs.

Our study demonstrates that clinicians use a series of heuristics following bounded rationality principles while not following a stepwise method to make diagnostic decisions. The use of heuristics was noted in our study to be highly intrinsic to the decision making process and simply negating it will only move DCPGs away from widespread implementation. Clinical reasoning cannot be interpreted as an autonomous set of rules, but as bounded rationality that is highly influenced by its surrounding environment. This finding not only has a say in the presentation of the DCPGs, but has a direct impact on evidence based education for future physicians. Nevertheless, to get this to a level of incorporation into DCPGs, further studies are needed to confirm, the generalizability as well as the impact on the outcome of patients when one uses heuristics as opposed to just guidelines. Assuming that physicians rely on bounded rationality, different methods can ease out the process. Research efforts to develop algorithms that integrate formal research knowledge with tacit knowledge should be developed allowing clinicians to keep using heuristics on a patient basis. Future research should focus on strategies to reduce the most common heuristics biases without creating cognitive overload. For example, a recent study showed the use of visuals generated from real time electronic health record data in facilitating the clinical decision making process [29].

Thus, an electronic health record system that provides a real time data analysis of the previously treated patients, in terms of their outcomes could assist in making heuristic decisions which also have their roots in past experiences. Such assistance could help in overcoming the subjective bias, a component of heuristics judgment, while also helping the clinician make use of real time evidence of their own data. Although while using these computational assistants, physicians need to derive initial hypothesis and to use their reasoning to input appropriate data [41]. Incorporation of such tools in real practice, as well as in the medical curriculum, will help in streamlining the process of heuristics in clinical practice.

Although we understand the limitations of the complex yet comprehensive guideline structure that proposes to include heuristics, we believe this new guideline structure would be received with lot of interest and would also find more usage than the current concept. The way of communicating DCPGs should be based on extended cognition [42] mechanisms that are compliant with our cognitive characteristics and needs rather than (wrongly) supposing that people have an unlimited amount of cognitive ability. Merging heuristics with its biases to the generalization of DCPGs is an urging issue in medical decision making.

## Supporting Information

List S1 (0.03 MB DOC)
