# Supplementary material for: Clinical Reasoning in the Real World Is Mediated by Bounded Rationality: Implications for Diagnostic Clinical Practice Guidelines
Source: PLoS One. 2010 Apr 20;5(4):e10265. doi: 10.1371/journal.pone.0010265 (PMC2857648; doi:10.1371/journal.pone.0010265)
Supplement: List S1 — (0.03 MB DOC) [file pone.0010265.s001.doc]

#### List S1: Definitions

- Hypothesis: a group of clinical signs and/or symptoms that constitutes a temporary diagnosis or an initial therapeutic plan.
- Heuristic: a set of rules used either in the attempt to find an initial hypothesis (general heuristic) or to prove that an existing hypothesis is the best diagnostic or therapeutic decision (hypothesis -specific heuristic).
- Contradiction: a failure of an attempt to prove that an existing hypothesis is the best diagnostic or therapeutic decision.
- Refutation of a contradiction occurs when a contradiction is refused, thus reinforcing the initial hypothesis.
- Automation: the process where a physician follows a heuristic until one of the rules is contradicted, and then followed by an attempt to refute the contradiction.
- Saturation: occurs when the number of attempts to prove the hypothesis satisfies the physician while all existing contradictions have been falsified.
- Decision: a point in the clinical reasoning process when the physician reaches saturation in relation to an anchor.
- Suspension: a point when the clinical reasoning is stopped while waiting for more information, with the decision being delayed.
